# Medical Devices and Real-World Data: Can We Improve Surveillance? Comment on "Quality and Utility of European Cardiovascular and Orthopaedic Registries for the Regulatory Evaluation of Medical Device Safety and Performance Across the Implant Lifecycle: A Systematic Review"

**DOI:** 10.34172/ijhpm.9557

**Published:** 2026-04-11

**Authors:** Marta Alcalde-Herraiz, Daniel Prieto-Alhambra

**Affiliations:** ^1^Health Data Sciences, Botnar Research Centre, University of Oxford, Oxford, UK.; ^2^Department of Medical Informatics, Erasmus MC University, Rotterdam, The Netherlands.

**Keywords:** Implantable Medical Devices, Observational Data, Real-World Evidence, Federated Analytics, Post-marketing Surveillance, Regulatory Decision-Making

## Abstract

Hoogervorst et al systematically reviewed European cardiovascular and orthopaedic medical device registries to assess their preparedness for regulatory decision-making. The authors found high heterogeneity between data sources, limited transparency, and incomplete patient/procedure data, hindering cross-registry comparisons and regulatory reliability. Despite these limitations, registries remain essential for post-marketing surveillance, as exemplified by the case of "Metal on Metal" hip implants. In this commentary, we highlight emerging or ongoing initiatives focused on improving real-world evidence for medical devices, such as the UK’s Medical Devices Outcomes Registry (MDOR), which seeks to address current limitations by developing a centralised database with linkage to electronic health records (EHRs). Parallel initiatives, including Sentinel, Data Analytics and Real-World Interrogation Network (DARWIN EU^®^), National Evaluation System for Health Technology (NEST), and Guidance and Tools for Real-World Evidence Generation and Use for Decision-Making in Europe (GREG), seek to strengthen real-world evidence through common data models (CDMs) and federated analytics. Specifically, NEST and GREG focus on enhancing real-world data methods and guidelines for medical devices and drug-device combinations. Overall, all these initiatives represent major progress towards more robust and transparent systems for medical device surveillance.

 In an era where real-world data are increasingly used to inform European regulatory decision-making, the systematic review conducted by Hoogervorst et al^1^ is crucial to understand the readiness of existing medical device registries to meet this demand. Building on their findings, this commentary aims to highlight existing initiatives and methodological frameworks that address the identified concerns in transparency, standardisation, and data completeness. Additionally, we further discuss how linkage of registries with electronic health records (EHR) and federated analytics can enrich and reshape medical devices post-marketing surveillance.

 Hoogervorst et al conducted a systematic review of European cardiovascular and orthopaedic registries with the aim of assessing their structural, methodological, and data quality characteristics. The study concluded that most of the identified registries lacked sufficient public transparency (in terms of structural and methodological characteristics), had inconsistent definitions of outliers and outcomes, and heterogeneous follow-up durations. In the authors’ opinion, all these points hindered their use for the study of device performance across databases. Additionally, the evaluated registries did not report patient/procedure-level completeness, or it was less than 95%, which is the minimum required by the International Medical Device Regulators Forum to inform regulatory decisions. Overall, the authors argued that such high heterogeneity and lack of transparency undermined the usefulness of the identified device registries for benchmarking and regulatory surveillance.

 Although the authors focused only on cardiovascular and orthopaedic registries, previous studies have also identified, described, and compared other types of registries.^2,3^ The interest in registries arises from the fact that they are not only a rich source of granular high-quality medical device data, but also, they have the potential to serve as key tools for post-marketing surveillance. In fact, some registries have played a fundamental role in the identification and evaluation of important safety concerns. The most famous example is the case of the “Metal on Metal” hip implants, found to have much higher revision rates than other hip implants, despite being originally developed as a more durable alternative.^4^ This concern was first raised by national registries in Europe through early outlier identification in revision rates.

 As highlighted by Hoogervorst et al and illustrated by the example of “Metal on Metal” hip implants, registries can be a very valuable resource to monitor long-term device performance, as well as allowing the benchmarking of different devices by comparing brands, models, and combinations. However, one of the current limitations of implant-based device registries is their limited scope, typically constrained to a clinical area (eg, orthopaedics) or anatomical region. A promising initiative that will overcome this is the UK-based Medical Devices Outcomes Registry (MDOR)^5^ (Figure). This new national registry aims to systematically track all high-risk medical devices implanted in England, and to link patient-level data to routinely collected EHR. MDOR will therefore constitute a centralised, patient-level registry to support the surveillance of a wide range of devices and implants. Importantly, the consistent collection of data in one centralised registry will overcome many of the inconsistencies flagged by Hoogervorst and colleagues. This dataset and others with a similar nature could change the post-authorisation surveillance paradigm for medical devices in the UK and internationally.

**Figure:**
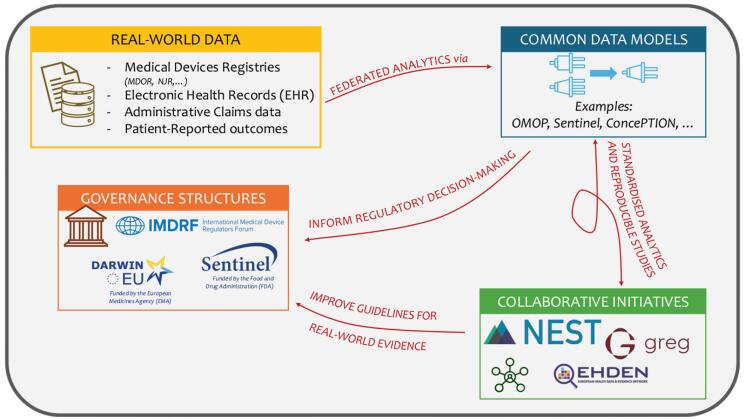
Illustration Summarising the Different Types of Real-World Data, Common Data Models, Collaborative Initiatives and Governance Structures, and the Interconnexion Between All These Different Elements. Abbreviations: MDOR, Medical Devices Outcomes Registry; NJR, National Joint Registry; OMOP, Observational Medical Outcomes Partnership; NEST, National Evaluation System for Health Technology; GREG, Guidance and Tools for Real-World Evidence Generation and Use for Decision-Making in Europe; EHDEN, European Health Data and Evidence Network; IMDR, International Medical Device Regulators Forum.

 Additionally, the linkage of MDOR to National Health Service (NHS) longitudinal EHR data will facilitate a consistent follow-up and the homogeneous definition of multiple potential outcomes. Such linkage allows for the holistic assessment of patient health, beyond the anatomical or therapeutic area treated by the device under evaluation. The benefits of linking registry data with hospital records for the study of diverse outcomes have been clearly demonstrated. For example, in a study conducted by Prats-Uribe et al^6^ the linkage of UK National Joint Registry data with routinely collected data from NHS hospital inpatient records allowed for the replication of a previous surgical randomised controlled trial: the Total or Partial Knee Arthroplasty Trial (TOPKAT). First, the authors successfully illustrated the validity of the data and proposed causal inference methods to replicate TOPKAT in terms of orthopaedic outcomes, including short-term patient-reported outcomes and long-term revision risks. Secondly, Prats-Uribe et al used their linked registry-EHR data to expand the trial findings and study additional outcomes, including cardiovascular and thromboembolic events, and to include patients not eligible for TOPKAT.

 Parallel to the increasing availability of registries, EHRs and claims alone have also been used for similar purposes. Their main advantages include their low cost (as they exist without a need for primary data collection) and wider availability. In line with the example above, Burn et al^7^ used claims and EHR (without registry linkage) exclusively to replicate the TOPKAT trial, reaching similar conclusions to the study using linked orthopaedic registry data.

 Routinely collected EHR are already playing a key role in the assessment of post-marketing surveillance of medicines and vaccines. For example, the US Food and Drug Administration’s Sentinel^8^ has established a network of EHR linked to insurance claims data to inform medical product surveillance, safety assessments, and regulatory discussions^9^ (Figure). In Europe, the European Medicines Agency (EMA) launched the Data Analytics and Real-World Interrogation Network (DARWIN EU^®^; https://www.darwin-eu.org/)^10^ in 2022 to deliver real-world evidence on medicines and vaccines for the European Medicines Regulatory Network, including (to date) a network of more than 40 databases with primary care data, hospital records, and nationwide registries.^11^ Since 2022, approximately 110 studies have been delivered, supporting labelling changes, informing regulatory reviews of medicines, and enhancing preparedness for public health emergencies.

 However, EHR and claims data have inherent limitations that have to be accounted for, especially when used for medical devices surveillance. Inconsistently recorded device data, fragmented healthcare (as patients may receive care from multiple systems), and a lack of data standardisation between different centres, may limit EHR capability to assess long-term outcomes and conduct multi-centre studies. On the other hand, claims data are restricted to insured populations, and are primarily collected for billing purposes, which can lead to underreporting of mild/moderate symptoms. Furthermore, EHR and claims often omit critical details like the unique device identifier and information of operating surgeons. This limits the granularity of the analyses and hinders accounting for learning-curve effects, despite their well-established impact on the performance of implantable devices and procedures.

 The lack of interoperability across databases is not unique to EHR; as highlighted by Hoogervorst et al, registries face the same challenge. Federated analyses based on the use of a common data model (CDM) have been proposed as a solution to enable efficient and reproducible analytics without transferring patient-level data^12^ (Figure). DARWIN EU^®^ data partners have all been mapped to the Observational Medical Outcomes Partnership (OMOP) CDM, therefore enabling standardised analytics and homogeneous definitions. Similar methods and the same CDM were used by the European Health Data and Evidence Network (EHDEN) public-private partnership, which led to the creation of a network of over 200 databases mapped to the OMOP CDM from 30 different European countries.^13^ EHDEN recently published the largest real-world evidence study to date, including over 50 databases from 18 European countries and the US for the study of drug shortages internationally.^14^

 Despite DARWIN EU^®^ and the mentioned EHDEN study demonstrate the scalability and potential of CDM-based federated analytics, standardised analytics also present important challenges. Data mapping can be complex, often requiring the transformation of free text or country-specific concepts into standardised vocabularies, which may result in information and granularity loss. Importantly, clinical practice may vary across data sources and countries. Therefore, even when the same analytics can be executed in different databases, careful consideration of the underlying clinical context is essential.

 Although previous initiatives focused on medicines and vaccines, more recent ones aim to expand and improve real-world evidence and federated analytics for medical devices. Since 2016, the National Evaluation System for Health Technology (NEST; https://nestcc.org/) has been established as an active national evaluation system for medical devices using real-world data in the US (Figure). Through the execution of clinically and methodologically relevant use cases, NEST has contributed to advances in data quality, methods research, choice of study designs, and data quality guidelines for medical device studies using real-world data.^15^ More recently, the Innovative Health Initiative Joint Undertaking funded the Guidance and Tools for Real-World Evidence Generation and Use for Decision-Making in Europe (GREG; https://ihi-greg.eu/) project to test, improve, and co-create guidance and tools for the generation and use of real-world evidence for the development and evaluation of medicines and devices in Europe. This 5-year initiative has a specific work package dedicated to the study of medical devices and drug-device combinations, and a separate one focused on the mapping and improvement of medicines and medical device data sources to accelerate real-world evidence generation. With this and other similar initiatives on the horizon, there is growing optimism that the barriers described by Hoogervorst et al will be overcome.

 Overall, while registries have historically been the primary resource for medical device post-marketing surveillance, the findings by Hoogervorst et al highlight the need for improving their quality, transparency, and interoperability. Building on these observations, this commentary emphasises that the future of medical device surveillance will likely depend on the integration of registries with other types of routinely collected healthcare data, including EHRs or administrative claims databases, alongside the adoption of CDMs and federated analytics. These complementary approaches have the potential to address many of the current limitations by enhancing data completeness, enabling consistent outcome definitions, and supporting transparent and reproducible analysis across different healthcare settings. Ultimately, all these efforts will not only strengthen the regulatory utility of real-world evidence for medical device post-marketing surveillance, but will also contribute to improving patient care and health outcomes.

## Acknowledgements

 Marta Alcalde-Herraiz PhD studies are funded by the Clarendon Fund and Wolfson Derek Boyd scholarship. Daniel Prieto-Alhambra’s research is supported by the National Institute for Health and Care Research (NIHR) Oxford Biomedical Research Centre (BRC).

## Disclosure of artificial intelligence (AI) use

 Not applicable.

## Disclaimer

 Funding sources were not involved in study design, data collection, data analysis, interpretation of data, writing of the study for publication, and in the decision to submit the article for publication. The views expressed are those of the author(s) and not necessarily those of the NIHR, the Department of Health and Social Care or the Clarendon Fund and Wolfson Derek Boyd Scholarship.

## Ethical issues

 Not applicable.

## Conflicts of interest

 Daniel Prieto-Alhambra’s research group from the University of Oxford has received research grants from the Innovative Medicines Initiative, Gilead Science, Theramex, EMA, and UCB Biopharma, none of which related to this Article. Daniel Prieto-Alhambra’s research group has also received research grants from the EMA. Daniel Prieto-Alhambra sits in the board of the EHDEN Foundation. Marta Alcalde-Herraiz declares no conflicts of interest.
